# Freeze-Dried *K*-Carrageenan/Chitosan Polyelectrolyte Complex-Based Insert: A Novel Intranasal Delivery System for Sumatriptan Succinate

**Published:** 2018

**Authors:** Sonia Alavi, Seyed Alireza Mortazavi

**Affiliations:** *Department of Pharmaceutics, School of Pharmacy, Shahid Beheshti University of Medical Sciences, Tehran, Iran. *

**Keywords:** Sumatriptan succinate, *k*-carrageenan /chitosan polyelectrolyte complexes, Freeze-dried inserts, Water uptake ability, Mucoadhesion strength, *In-vitro* drug release, Nasal drug delivery

## Abstract

Intranasal route, ensuring suitable bioavailability of medicines under circumvention of the gastrointestinal degradation and hepatic first-pass elimination, has been a popular choice for drug delivery. Among nasal dosage forms, mucoadhesive solid inserts have been shown to resist mucociliary clearance and provide a prolonged nasal residence time. Hence, the purpose of this study was the preparation and characterization of nasal inserts composing of polyelectrolyte complexes (PECs) based on *k*-carrageenan (*k*-CA) and chitosan (CS) to boost therapeutic efficacy of sumatriptan succinate in the treatment of migraine headache. *k*-CA/CS PECs were developed in different molar ratios, subjected to lyophilization in small inserts in the presence of sumatriptan succinate, and finally investigated for water uptake ability, mucoadhesive potential, and drug release profile. The formation of PEC between the two polymers was affirmed by Fourier transform infrared spectroscopy (FTIR). Based on the results, it was revealed that the polyanion/polycation molar ratio plays a critical role in modulating the characteristics of the inserts, and among all the formulations, the one comprising *k*-CA/CS PEC with molar ratio of (4:1), (*k*-CA/CS _(4:1)_), demonstrated the highest water uptake ability and mucoadhesive potential and provided a more controlled release of sumatriptan succinate. This study illustrates the potential of the lyophilized inserts based on the *k*-CA/CS PECs, especially *k*-CA/CS _(4:1)_, for efficient delivery of sumatriptan succinate via the nasal route of administration and suggests a potential therapeutic approach for the termination of migraine attacks.

## Introduction

Migraine is the most frequent neurological disorder, affecting approximately 16% of the worldwide population (1). This debilitating disease is chiefly characterized by recurrent episodes of unilateral throbbing headache, associated with neurological symptoms, including hypersensitivity to light (photophobia), sound (phonophobia), and smell, as well as impairment of autonomic, cognitive, motor, and emotional functions (2, 3). The complexity of pathophysiological processes, involved in migraine, hinders its definitive treatment; however, there are several classes of medications with different pharmacological mechanisms, which have been developed for its therapeutic management (4, 5). Sumatriptan was the first triptan, approved by US FDA in the early 1990s for the termination of migraine attacks. It exerts its pharmacological action by selectively binding to 5-hydroxytryptamine type-1B and -1D (5-HT1B/1D) receptors, leading to vasoconstriction of extensively dilated intracranial blood vessels and inhibition of the release of pro-inflammatory neuropeptides that have been found to play pivotal roles in migraine symptomatology (6, 7). Sumatriptan is currently available on the market in formulations, suitable for oral, subcutaneous, and intranasal administration. Although, a significant proportion of migraineur patients suffer moderate to severe gastrointestinal disturbances during their migraine attack, which can lead to erratic absorption of oral medications from the gastrointestinal tract, and thus make oral treatment unsatisfactory (8). Subcutaneous administration offers an alternative option to oral delivery; however, aversion or fear of injection and challenges of self-administration make subcutaneous treatment offensive to some patients. Aiming to surmount these obstacles, nasal administration of sumatriptan has acquired conspicuous popularity in recent years. Indeed, the relatively large surface area (~150 cm^2^), porous endothelial basement membrane, and high vascularity of the nasal mucosa ensure rapid and high systemic availability of the delivered therapeutics under circumvention of the hepatic “first-pass” elimination (9). However, mucociliary clearance in the nasal cavity that consists of a coordinated interaction between the mucus layer and the methachronal movement of cilia, tends to clear conventional dosage forms from the nasal cavity in approximately 15-20 min after administration, which exerts a negative influence on the bioavailability of their contents (9). In situ gelling mucoadhesive nasal insert has been recently proposed to overcome this challenge, and received a lot of research momentum (10, 11). This novel dosage form, which is developed by lyophilization, comprises a sponge-like hydrophilic polymer matrix, in which the cargo is embedded. Upon getting in contact with the nasal mucosa, the highly porous lyophilized insert rapidly withdraws water and forms a gel structure, which decreases the ciliary clearance rate and serves as a depot, providing extended release of its payload over a period of several hours (12).

The use of mucoadhesive polymers in this formulation can guarantee an intimate contact with the nasal mucosa, which yields a steeper concentration gradient of the administered therapeutics across this layer, and thus augments their absorption and bioavailability (13). Besides, other advantages, including ease of administration, accuracy of dosing, and reduced feeling of foreign body sensation also accompany this formulation approach. A number of synthetic or natural polymers have been employed for the fabrication of nasal inserts. However, relatively little work has been carried out on the development of inserts based on polyelectrolyte complexes (PECs), which are the products of electrostatic interactions between oppositely charged polymers, and have been found to exhibit better functional properties compared to their individual components.

Chitosan (CS) and carrageenan (CA) are two naturally occurring biopolymers, which are widely exploited in the pharmaceutical field. CS, a natural derivative of chitin, is a cationic polymer, consisting of randomly distributed β-(1-4)-linked N-acetyl-d-glucosamine and d-glucosamine (14). CAs, anionic sulphated polymers of galactose and anhydrogalactose, are extracted from marine red algae and have three main fractions, namely iota (*i*-), kappa (*k*-), and lambda (λ-).

It is worth to mention that the first two types have the ability of gel formation, whereas λ-CA is a thickener, not a gelling entity (15).

Therefore, the purpose of the present study is to develop lyophilized nasal inserts, composed of CS and *k*-CA for instranasal delivery of sumtriptan succinate and to characterize them in terms of water uptake ability, mucoadhesive potential, and release behavior to determine their potential for future clinical translation.

## Experimental

*Materials*

Sumatriptan succinate (purity > 98 %) was kindly gifted by Damavand Darou Pharmaceutical company (Iran). CS (Mw 110, 000–150,000) commercial grade *k*-CA (*k*-CA; No. C-1013) consisting of predominantly *k*- and lesser amounts of λ-CA, and mannitol were all purchased from Sigma-Aldrich (USA). Water used in the experiments was deionized and produced using a Milli-Q water purification system (Millipore, USA). All other chemicals were of analytical grade and supplied by Merck (Germany).

*Preparation of k-CA /CS complex nasal inserts*

*k*-CA/CS complex nasal inserts were prepared by ionic complexation, via electrostatic interactions between the negatively charged sulfate groups of *k*-CA and the positively charged amino groups of CS. 

Briefly, *k*-CA was dissolved in water and CS was dissolved in acetate buffer pH 5.0, to obtain solutions of 2 % (w/v) concentration. The *k*-CA solution was then added to the CS solution in five different molar ratios (4:1, 2:1, 1:1, 1:2, and 1:4; mol *k*-CA /mol CS), under magnetic stirring for 24 h at room temperature. The precipitates were separated using centrifugation at a speed of 10,000 rpm for 10 min (Hermle Z320, Germany), rinsed with water, and then homogenized at 17,500 rpm for 5 min with an Ultra-Turrax T 25 basic homogenizer (IKA-Werke, Germany). Subsequently, an aqueous solution of mannitol, a common bulking agent, was prepared and added slowly to the homogenized dispersed complexes with gentle stirring to achieve a theoretical complex/mannitol ratio of 9:1 (w/w), and then the resultant suspensions were stored at 4 °C overnight to get rid of air bubbles. 

Finally, PEC gels were transferred into polypropylene micro-centrifuge tubes, (Eppendorf, Germany), frozen at −20 °C for 12 h, and then subjected to freeze-drying by an Alpha 1–2 LD plus Freeze Dryer (Martin Christ, Germany) at −55 to −60 °C and a vacuum level of 0.05 mbar for 24 h (16). 

When preparing loaded inserts, the procedure was the same as above, but sumatriptan succinate was dissolved in water and added to the solutions of PECs that allowed preparing inserts with three different drug/complex weight ratios (1:4, 1.5: 4, and 2: 4).

Besides, loaded inserts, comprised of only mannitol and sumatriptan succinate, were also prepared as control formulations for *in-vitro* release studies.

*Physicochemical characterization*

The prepared inserts were investigated in terms of Fourier transform infrared (FTIR) spectroscopy, drug content, water uptake ability, mucoadhesion potential, *in-vitro* release profile, and stability.

*Fourier transform infrared (FTIR) spectroscopy*

The mid-IR spectra (4000 – 400 cm ^−1^) of the powder samples (*k*-CA, CS, and the PEC containing equal amounts of both polymers) were recorded in the absorbance mode at a spectral resolution of 4 cm ^−1^ via a Nicolet Magna 550 spectrometer (Thermo Nicolet, USA), having a germanium-coated potassium bromide (KBr) beam splitter and a deuterated triglycine sulphate (DTGS) detector.

*Drug content*

The loaded inserts (n = 10) were individually weighed and triturated to get homogeneous mixture. A quantity of powder, equivalent to the mass of one insert (30.0 mg), was extracted in 100.0 mL of ethyl alcohol and filtered through a filter paper (pore size 0.45 μm, Millipore, USA). The drug content was measured by UV spectroscopy (UV-mini 1240, Shimadzu, Japan) at λ max = 226 nm through a reference to an appropriate calibration curve of the sumatriptan succinate (R^2^ = 0.998).

*Water uptake studies*

A sponge (6 cm×6 cm×2 cm) was completely soaked in the hydration medium (phosphate buffers of pH 2, 5.5, and 7.4) and put on a petri dish, which was filled with the same medium to a 1 cm height, to be soaked over the study period. Circular filter paper (Whatman^®^, USA) was also soaked in the medium and laid on top of the sponge. 

Following the equilibration period of 30 min, unloaded inserts (with various ratios of *k*-CA and CS) were accurately weighed (100.0 mg) and positioned on the filter paper (10).

Water uptake ability of the inserts was then calculated, as their weight increase after 6 h, using equation (1), where W_T_ represents the total weight of hydrated formulation and moist filter paper, W_P_ is the weight of moist filter paper, and W_I_ represents the initial weight of the formulation (10). 

$\mathrm{\% Water uptake}(\mathrm{\%WU})=\frac{\left(W_{T}-W_{P}-W_{I}\right)}{W_{I}}\times 100$

Equ. 1

The effect of drug incorporation on the water uptake ability of the loaded inserts was also investigated at pH 5.5, which is close to the physiologically normal nasal pH.

*Mucosadhesion studies*

*Preparation of the model mucosa (test surface)*

The model mucosal membrane, utilized in this study, was sheep nasal mucosa. For this purpose, the septum and turbinates of the sheep’s nose, obtained from a local slaughter house, were fully exposed via a longitudinal cut along the nose. The mucosal membrane was carefully freed from the underlying cartilage and bone, and then frozen at -20 °C until required to prevent muscle contraction, therefore ensuring the flat and consistent surface, necessary for this type of adhesion experiment. When required for use, the tissue was permitted to thaw at 4 °C, chopped approximately into 4 cm length pieces, which were then washed gently with phosphate buffer (pH 5.5) to remove any residual nasal contents, and finally employed for the study. 

*Ex-vivo mucosaadhesion force*

**Figure. 1:**
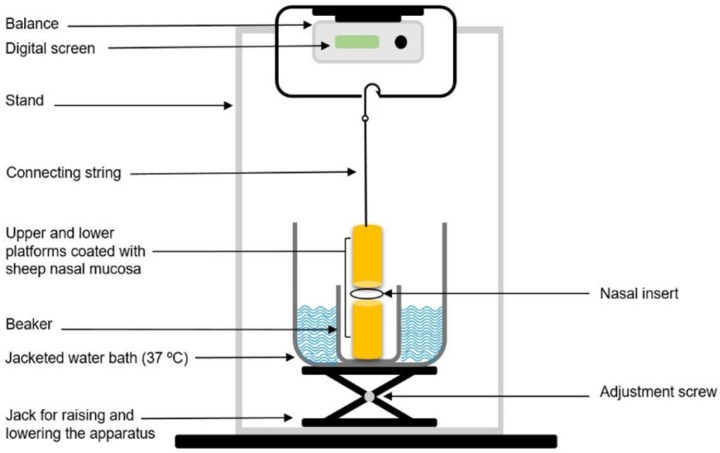
Schematic diagram of the apparatus used for assessing the mucoadhesive strength of the inserts

In order to investigate the mucoadhesive behavior of the inserts, a lab-fabricated apparatus, which was mainly similar to those described in previous studies, was utilized (Figure 1) (17, 18). The upper stationary platform of the apparatus was connected to a balance, determining the force required to detach the insert from the mucosa. The test cell was filled with phosphate buffer (pH 5.5), and kept at 37 °C to simulate the nasal physiological environment. The pieces of the nasal mucosa were mounted and fixed on the two cylindrical platforms (mucosal side upwards) with cyanoacrylate adhesive and left to equilibrate in this medium for 2 min. The inserts were separately positioned between the two mucosa-covered platforms, and maintained in place for 5 min. A continuous rising force of 0.1 g/sec was then applied on the adhesive joint developed between the insert and the mucosa, through slowly lowering the lower platform, to break the contact between these elements and the detachment force determined was recorded.

*In-vitro release studies*

The *in-vitro* release of sumatriptan succinate from the loaded inserts was investigated using a lab-fabricated assembly, adapted in order to simulate the physiologic status of the nasal mucosa (19). The lower end of a polypropylene tube (approximately 3.5 cm in inner diameter) was tightly closed with a filter paper (pore size 0.45 μm, Millipore, USA). The tube was vertically positioned into a release medium container, which was filled with 20.0 mL phosphate buffer (pH 5.5), and then adjusted so that the filter paper was wetted, but not submersed in the release medium. The loaded inserts (drug/complex weight ratio of 1.5:4) were located on the filter surface, and the whole assembly was closed with Parafilm® “M” (American National Can Company, USA) to prevent evaporation of the medium during the experiment and to establish a constant relative humidity to which the inserts were exposed to. The drug release study was carried out under 75 rpm magnetic stirring at 37 °C up to 6 h. At specific time points, aliquots (500 µL) were taken out from the release medium and the same amount was replenished with the fresh medium to ensure the maintenance of sink condition. The amount of drug released was assayed spectrophotometrically as described for the determination of drug content.

*Stability studies*

Physical stability studies were carried out based on International Conference on Harmonization (ICH) guidelines (20). The loaded inserts were placed in polyethylene bottles and stored in a desiccator containing silica gel, which was maintained at 40 °C for 3 months. At regular time intervals, the inserts were investigated for any change in their appearance characteristics, such as shape and color, and drug content. 

*Statistical analysis*

All the experiments were carried out in triplicate, and the results were expressed as mean value ± standard deviation (SD). Statistical analysis was performed via the analysis of variance (ANOVA) by means of the SPSS 17.0 software, and the differences of P less than 0.05 were interpreted as denoting statistical significance.

## Results and Discussion

As mentioned earlier, in situ gelling mucoadhesive inserts have recently presented a new opportunity for nasal delivery of therapeutics. Because of the rather limited studies conducted on them, especially those comprising of PECs, in this study attempts were made for the development of inserts, composed of CS and *k*-CA, and study of their potential for nasal delivery of sumtriptan succinate.

*Fourier transform infrared (FTIR) spectroscopy*

**Figure 2:**
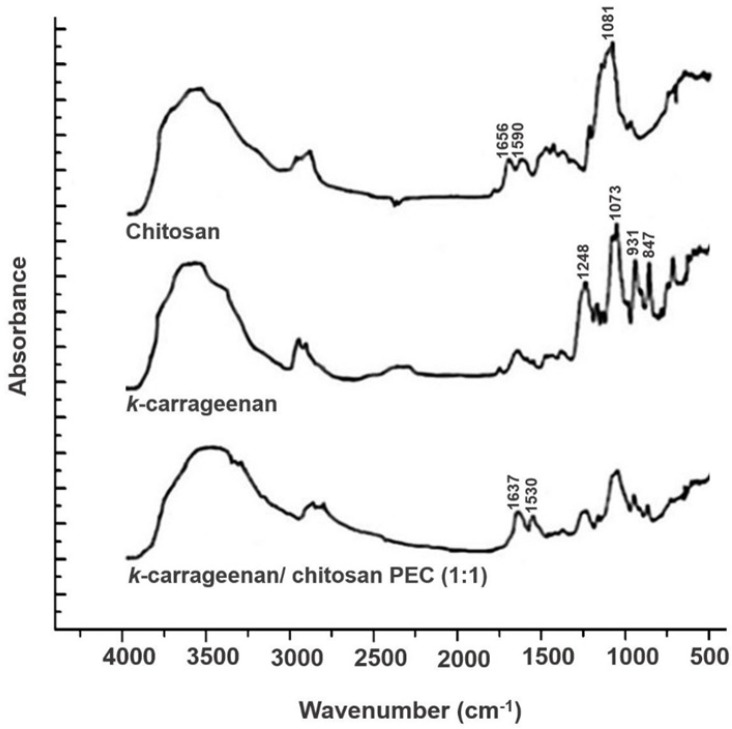
FTIR spectra of *k*-CA, CS, and the PEC containing equal amounts of both polymers scanned over the wavenumber range of 4000 – 400 cm^-1^

Figure 2 exhibits the FTIR spectra, achieved from the analysis of *k*-CA, CS, and the PEC containing equal amounts of both polymers,* k*-CA/CS _(1:1)_. As expected, *k*-CA showed several peaks, among which four main peaks were prominent, including a peak at 1248 cm^−1^ attributed to the ester sulfate groups, a peak at 1073 cm^−1^ ascribed to the glycosidic linkage, and peaks at 931 and 847 cm^−1^ , corresponding to the 3,6-anhydrogalactose and galactose-4-sulfate, respectively (21, 22). 

The FTIR analysis of CS exhibited characteristic peaks of amide I and II groups at 1656 and 1590 cm^−1^, respectively. Besides, a peak which is assigned with the glycosidic bonds was also identified at 1081 cm^−1^(23).

The FTIR spectrum of the PEC, obtained upon interactions of *k*-CA and CS, demonstrated a new absorption peak at 1530 cm^−1^, related to NH^+3^ groups, which is not present in the spectra of the two polymers. Moreover, it was observed that amide peaks of CS were coalesced into a singlet peak at 1637 cm^−1^, and typical peaks of *k*-CA were still identified, but presented smaller intensities. These results evidence the successful development of PEC via electrostatic interactions between the oppositely charged polymers.

*Drug content*

The drug content of all the formulations (*k*-CA/CS _(4:1)_ to *k*-CA/CS _(1:4)_) was determined and the results are presented in Table 1. According to the results, the drug content was found to be in the range of 98.00 - 101.00 (%), indicating uniform and relatively complete drug loading in all the formulations.

**Table 1:** The composition and characteristics of the *k*-CA/CS PEC-based inserts; data are represented as mean ± SD (n = 3)

**Formulation**	***k*** **-CA:CS molar ratio**	**Appearance**	**Drug content (%)**
*k*-CA/CS _(4:1)_	4:1	White sponge-like solid	98.14 ± 0.71
*k*-CA/CS _(2:1)_	2:1	White sponge-like solid	98.69 ± 0.57
*k*-CA/CS _(1:1)_	1:1	White sponge-like solid	99.17 ± 0.26
*k*-CA/CS _(1:2)_	1:2	White sponge-like solid	100.92 ± 0.49
*k*-CA/CS _(1:4)_	1:4	White sponge-like solid	99.21 ± 0.30

*Water uptake studies*

**Figure. 3:**
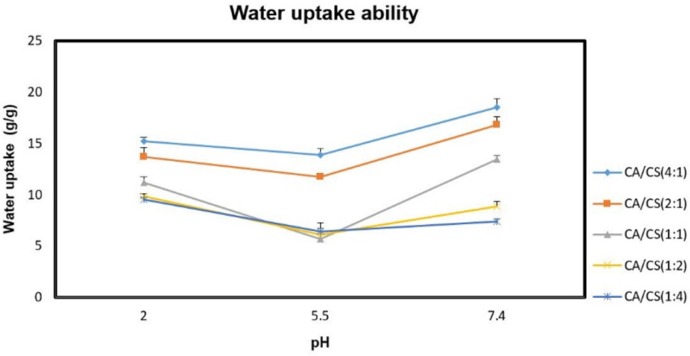
Water uptake ability of the inserts under different pH conditions after 6 h; data are represented as mean ± SD (n = 3)

The water uptake ability of the inserts is an essential step for their transition into the gel state and adhesion to the mucosal tissue, which is attributed to the presence of hydrophilic functional moieties, like OH, COOH, and OSO_3_H in their polymeric structures. The hydration of these moieties leads to water entry into the polymer matrix, resulting in expansion and ordering of the polymer chains, which continues until the osmotic forces of the functional moieties are balanced by the elastic restoring forces of the network structure (24). The water uptake ability of the unloaded inserts under various pH conditions was presented in Figure 3. Regarding the results, water uptake ability of the *k*-CA/CS PECs was greatly affected by polyanion/polycation molar ratio and pH of the medium, which is consistent with previous researches (16, 19).

As can be seen, higher amounts of *k*-CA in the PECs allowed for a greater water uptake ability. This can be probably ascribed to the ionic nature of *k*-CA, able to favor a greater entry of water into the system, whereas the weak aqueous solubility of CS restricts the water absorption of the formulations. Besides, water uptake ability of all the PECs investigated was lower at pH 5.5 than at pH 2.0 and 7.4 conditions (*P* < 0.05). This is due to the fact that when the PECs are hydrated within the pKa interval of the two polymers, the electrostatic interactions between opposite charges in their structure undergo only minimal or no modification, leading to a less water uptake. While, the presence of a large excess of free charges inside their structure at pH 2.0 and 7.4 provides great water uptake. 

**Figure 4:**
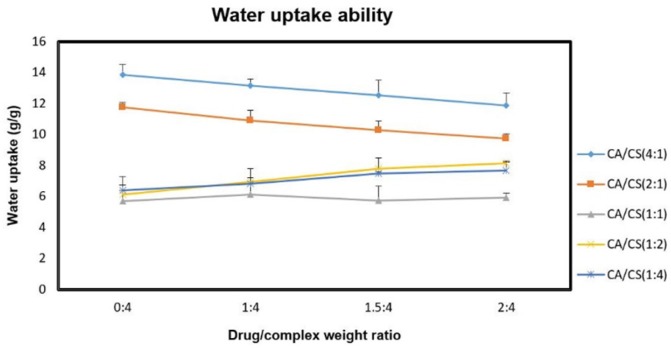
Water uptake ability of the differently loaded (drug/complex weight ratios of 1:4, 1.5:4, and 2:4) inserts at pH 5.5 after 6 h; data are represented as mean ± SD (n = 3).

The effect of sumatriptan succinate on the water absorption of the formulations was also evaluated at pH 5.5 (Figure 4). 

Regarding the results, there was a concentration-dependent decrease in water uptake ability of the inserts containing higher amounts of *k*-CA with increasing concentrations of drug (*P* < 0.05), which can be justified by the presence of the tertiary amine (pKa 9. 63) and the sulfonamide (pKa > 12) groups of sumatriptan (25), capable of interacting with free negative charges (*k*-CA sulfate groups) in the PEC structure during the loading process, hence resulting in the development of less hydratable complex (10). While, increasing concentrations of drug within the inserts containing higher amounts of CS enhanced their water uptake ability (*P* < 0.05), which can be related to the repulsive electrostatic interactions between the drug molecules and free positively charged amino groups of CS during the loading process, leading to the formation of more hydratable complex. 

*Mucosadhesion studies*

The mucoadhesion is an important strategy to prolong the residence time of drug delivery systems in the nasal cavity in order to provide extended drug delivery. 

Upon nasal administration and getting contact with the mucosal surface, lyophilized insert absorbs water from the surrounding moist environment and swells to form a gel structure, capable of interacting with the mucus layer through physical entanglement and secondary interactions (i.e. hydrogen bonding and Van der Waals forces), which allows for a prolonged drug delivery (26). At pH 5.5, complete ionization of sialic and sulfonic acid substructures of the mucin glycoproteins confers negative charges to the nasal mucusa (27). 

**Figure 5:**
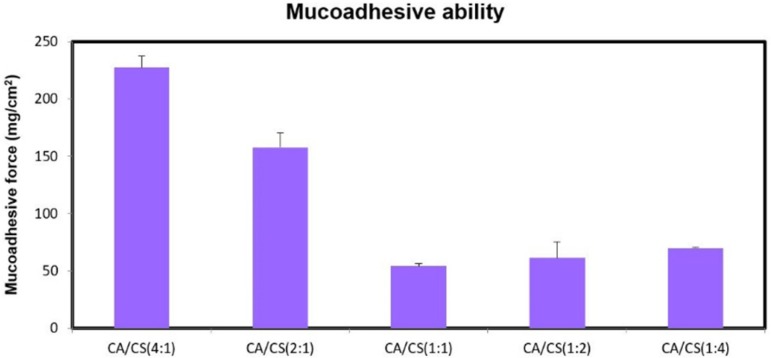
Mucoadhesive strength of the *k*-CA/CS PEC-based inserts at pH 5.5

While, despite the presence of negative charges on *k*-CA chains (pKa of about 2.0), attributed to the ionization of the sulfate groups, and positive charges on CS chains (pKa of 6.3), ascribed to the ionization of the amino groups, the higher the proportion of *k*-CA, the greater was the mucoadhesion ability (*P* < 0.05) (Figure 5).

This can be mainly explained by the greater water uptake ability of *k*-CA compared to CS, allowing for a more efficient mobility of the polymeric chains and their physical entanglement with mucus.

*In-vitro release studies*

**Figure. 6:**
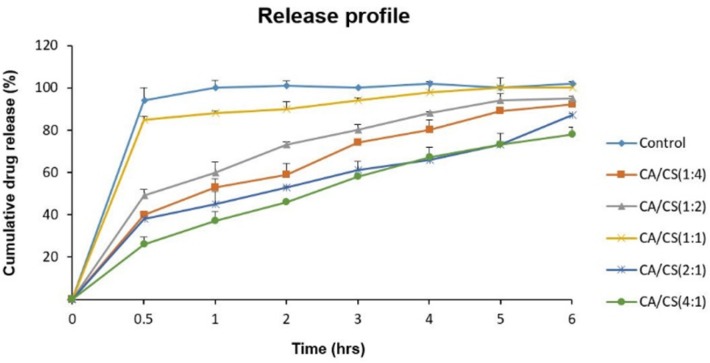
*In-vitro* release profiles of sumatriptan succinate from the control formulation and loaded (drug/complex weight ratios of 1.5:4) inserts at pH 5.5; data are represented as mean ± SD (n = 3).

The release profiles of sumatriptan succinate from the loaded inserts (drug/complex weight ratio of 1.5:4) at pH 5.5 are illustrated in Figure 6. As it is clear, despite an immediate release of sumatriptan succinate from the control formulation, which is explained by the high solubility and fast dissolution of mannitol, no significant burst release was observed from any of the PECs, showing their ability to control the release of their contents.

Regarding the results, formulations containing higher proportions of *k*-CA (*k*-CA/CS _(4:1)_ and *k*-CA/CS _(2:1)_) exhibited more controlled release of the drug (*P* < 0.05), which can be ascribed to the greater tendency of *k*-CA to uptake water compared to CS, leading to a higher polymeric chain mobility, and thus developing a more viscous network. 

Furthermore, electrostatic interactions of drug molecules with *k*-CA chains may also contribute to such a decrease in release from the PECs comprising of high *k*-CA contents. 

In contrast, the *k*-CA/CS_(1:1)_ complex demonstrated the highest drug release among all the PECs, which can be related to the high degree of electrostatic interactions between the oppositely charged polymers and the absence of or low amounts of free charges, preventing the ingress of water into the PEC and polymeric chain mobility, and thus giving rise to an easier release of the drug.

*Stability studies*

The physical stability of all the formulations (*k*-CA/CS _(4:1)_ to *k*-CA/CS _(1:4)_) was studied at 40 °C for a period of three months via their appearance characteristics and drug content. As illustrated in Table 2, appearance characteristics of all the PECs nearly remained unaltered, and their drug content did not significantly change, exhibiting their considerable stability over the period of study.

**Table 2:** The characteristics of the *k*-CA/CS PEC-based inserts stored at 40 °C for 3 months; data are represented as mean ± SD (n = 3)

**Formulation**	**Duration (months)**	**Appearance**	**Drug content (%)**
*k*-CA/CS _(4:1)_	1 2 3	No change No change No change	97.70 ± 0.14 97.23 ± 0.72 96.76 ± 0.29
*k*-CA/CS _(2:1)_	1 2 3	No change No change No change	98.17 ± 0.34 97.86 ± 0.23 97.05 ± 0.61
*k*-CA/CS _(1:1)_	1 2 3	No change No change No change	98.51 ± 0.27 97.67 ± 0.64 97.05 ± 0.82
*k*-CA/CS _(1:2)_	1 2 3	No change No change No change	100.15 ± 0.11 99.68 ± 0.51 99.13 ± 0.39
*k*-CA/CS _(1:4)_	1 2 3	No change No change No change	98.75 ± 0.25 98.11 ± 0.78 97.65 ± 0.15

## Conclusion

The current study focused on the development and characterization of *k*-CA/CS-based in situ gelling inserts for intranasal delivery of sumatriptan succinate. The studies presented here suggested that the polyanion/polycation molar ratio is capable of modulating the characteristics of the inserts, including water uptake ability, mucoadhesion behavior, and drug release profile. Regarding the results, among all the inserts, the formulation comprising *k*-CA/CS PEC with molar ratio of (4:1), (*k*-CA/CS _(4:1)_), exhibited the highest water uptake ability and mucoadhesion potential, meaning that greater entry of water into the inserts′ matrix increases the mobility of polymeric chains and allows for their more efficient interactions with the nasal mucosa. Furthermore, such a great tendency to uptake water resulting in the development of a more viscous network, along with electrostatic interactions of drug molecules with *k*-CA chains, provided a more controlled release of the embedded drug. Thus, the formulation comprising the *k*-CA/CS _(4:1)_ PEC with ideal mucoadhesion strength and controlled release pattern would be promising delivery system for sumatriptan succinate in the treatment of migraine attacks. However, intranasal absorption studies in animal models need to be warranted to derive the feasibility of this system to boost the therapeutic efficacy.
